# Dual‐Phase Enhanced CT‐Derived Radiomics Nomogram for Progression‐Free Survival Prediction in Stage IV Lung Adenocarcinoma

**DOI:** 10.1002/cam4.70473

**Published:** 2024-12-09

**Authors:** Haitao Sun, Zhaohui Peng, Guoyue Chen, Zhengjun Dai, Jian Yao, Peng Zhou

**Affiliations:** ^1^ Medical Imaging Center of Central Hospital Affiliated to Shandong First Medical University Jinan Shandong China; ^2^ Scientific Research Department of Huiying Medical Technology Co. Ltd Beijing China; ^3^ Medical Imaging Center of Shandong Provincial Hospital Affiliated to Shandong First Medical University Jinan Shandong China

**Keywords:** computed tomography, lung adenocarcinoma, nomogram, progression‐free survival, radiomics

## Abstract

**Purpose:**

The objective is to establish a radiomics nomogram (Rad‐nomogram) using dual‐phase enhanced computed tomography (DPE‐CT) for the prediction of progression‐free survival (PFS) in patients diagnosed with stage IV lung adenocarcinoma (ADC).

**Methods:**

From DPE‐CT scans, radiomic characteristics were retrieved from 133 patients diagnosed with stage IV lung ADC. Clinical data were analyzed using univariate and multivariate Cox regression analyses. The radiomics signature was combined with clinical features employing multivariate Cox analysis in order to develop a Rad‐nomogram. The predictive efficiency of the nomogram was evaluated using survival studies, such as Kaplan–Meier curves and Harrell's C‐index. The benefits and clinical utility of various models were compared using the net reclassification index (NRI), decision curve analysis (DCA), and integrated discrimination improvement (IDI).

**Results:**

In the test cohort, the C‐indexes for the clinical, artery, and vein phase CT models were 0.675, 0.691, and 0.678, respectively. The dual‐phase achieved a C‐index of 0.731, exceeding the CT model, while the developed nomogram reached a C‐index of 0.783. The Kaplan–Meier survival study classified patients into low‐risk and high‐risk groups related to PFS using the Rad‐nomogram (*p* < 0.05). The Rad‐nomogram demonstrated a greater net advantage when compared with clinical and Rad models, as indicated by positive values of the NRI and IDI (ranging from 11.6% to 52.6%, *p* < 0.05).

**Conclusion:**

The Rad‐nomogram, employing DPE‐CT scans, offers a promising approach to predict PFS in individuals diagnosed with stage IV lung ADC.

**Abbreviations**
ADC: adenocarcinoma
ALC: advanced lung cancer
CA199: carbohydrate antigen 199
CEA: carcinoembryonic antigen
DCA: decision curve analysis
DPE‐CT: dual‐phase enhanced computed tomography
EGFR: epidermal growth factor receptor
GLCM: gray‐level co‐occurrence matrix
GLDM: gray‐level dependence matrix
GLRLM: gray‐level run length matrix
GLSZM: gray‐level size zone matrix
ICC: intraclass correlation coefficients
IDI: integrated discrimination improvement
LASSO: least absolute shrinkage and selection operator
NGTDM: neighboring gray‐tone difference matrix
NRI: net reclassification index
NSCLC: Non‐small‐cell lung cancer
NSE: neuro‐specific enolase
OS: overall survival
PFS: progression‐free survival
SCLC: small‐cell lung cancer

## 1 Introduction

One of the most common types of cancer and the leading cause of cancer‐related mortality is non‐small‐cell lung cancer (NSCLC). Lung adenocarcinoma (ADC) is the most common histological subtype of NSCLC. Approximately 50% of lung ADC cases are identified at advanced stages (III or IV), which correlates with a dismal prognosis [1, 2]. Immune checkpoint inhibitors, antiangiogenic drug combinations, and molecularly targeted treatments have been the mainstays of recent systemic therapeutic developments for stage IV lung ADC. These treatment approaches have increased progression‐free survival (PFS) and substantially inhibited the course of the disease [3, 4].

The emergence of primary or acquired drug resistance, resulting from the adaptive and evolutionary capabilities of tumor cells, presents a significant challenge to therapeutic efficacy and patient outcomes [5, 6]. Computed tomography (CT) imaging is widely employed in clinical practice for diagnosing and monitoring the treatment of advanced lung cancer (ALC). As a result, a predictive approach using CT imaging to evaluate individual progression probabilities may offer essential insights for customizing therapeutic strategies and potentially improving patient outcomes.

Predicting treatment results for patients with ALC has drawn much interest to radiomics analysis. Based on 2D and 3D CT imaging, this method has proven effective in identifying high‐risk and low‐risk patients undergoing first‐line targeted therapy for advanced NSCLC with epidermal growth factor receptor (EGRF) mutations and forecasting overall survival (OS) [7, 8]. Furthermore, radiomics has shown efficacy in predicting OS and PFS in NSCLC patients receiving chemotherapy and immunotherapy as first‐line treatments [9, 10]. Recent research studies have demonstrated that radiomic characteristics from single‐phase enhanced CT scans can predict PFS in patients receiving immunotherapy for NSCLC and small‐cell lung cancer (SCLC) [11, 12]. Enhanced CT radiomics is more clinically effective than plain CT radiomics [13].

Further, radiomics based on multi‐phase enhanced CT images outperform single‐phase imaging in predicting gene mutations and histopathological subtypes of lung cancer [14, 15]. Despite these advancements, research on DPE‐CT‐based radiomics for predicting therapeutic efficacy in advanced NSCLC is still insufficient. This method may be beneficial for examining tumor heterogeneity, considering the significant tumor volumes and degeneration frequently observed in ALC. This approach could be particularly beneficial in analyzing tumor heterogeneity, given the large tumor volumes and degeneration often seen in ALC. Some studies have utilized arterial and venous phase CT radiomics to evaluate and compare immunotherapeutic efficacy in NSCLC, but research specifically on DPE‐CT radiomics is limited [16]. A previous research study indicates that DPE‐CT radiomics can effectively predict pathological characteristics [17].

Considering this, the current study aimed to create a nomogram that forecasts PFS in stage IV lung ADC by combining radiomics and clinical data from DPE‐CT. The main objective was to establish a model for personalized prediction of disease progression.

## 2 Materials and Methods

### 2.1 Patients and Demographics

This retrospective analysis examined patients diagnosed with stage IV lung ADC at our hospital between January 2021 and June 2023. The institutional review board evaluated and approved the study, providing a waiver for informed consent. Retrospective collection of various laboratory, clinicopathological, and imaging data was conducted. This included information on variables such as gender, age, hypertension, diabetes, smoking habits, emphysema, family history of cancer, as well as pathological and laboratory markers like Ki‐67, P53, gene mutation status, neuro‐specific enolase (NSE) serum levels, carcinoembryonic antigen (CEA), and carbohydrate antigen 199 (CA199). Alteration in EGFR, the Kirsten rat sarcoma viral oncogene, anaplastic lymphoma kinase, and other genes were among the assessed gene mutations. In ALC, high and low expression levels were identified using a P53 threshold value of 10% and a Ki‐67 cutoff value of 30% [18, 19, 20].

Patients were administered first‐line systemic treatments comprising molecularly targeted therapeutics, immune checkpoint inhibitors, chemotherapy, and anti‐angiogenic agents. Treatment efficacy was assessed via routine laboratory tests and enhanced chest CT scans performed at 4 to 12‐week intervals. The PFS was defined as the duration from the initiation of treatment until the confirmation of disease progression or the patient's mortality. The results were assessed according to the criteria established by RECIST 1.1 [21].

**FIGURE 1:**
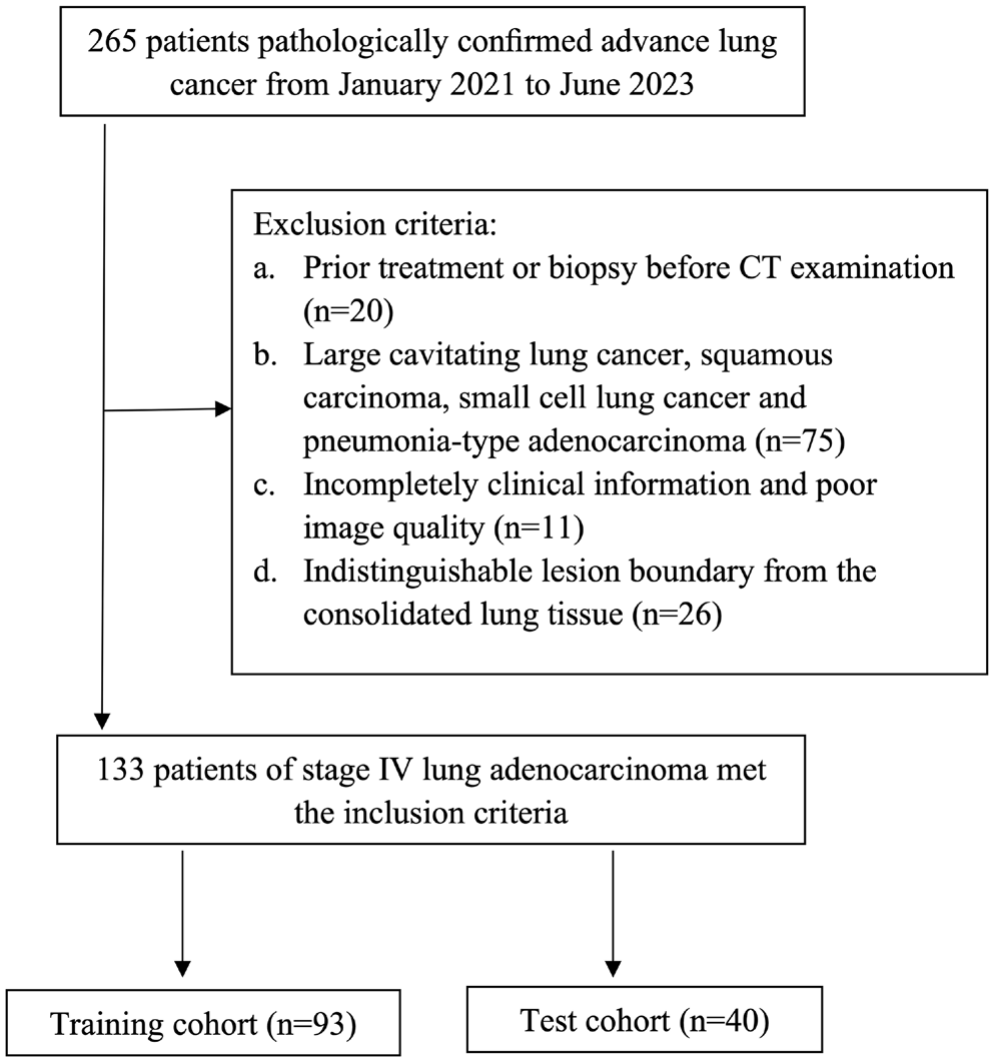
The flowchart for the selection of patients. CT, computed tomography.

The study's inclusion criteria were: [1] histopathologically verified stage IV lung ADC and [2] chest‐enhanced CT conducted before needle biopsy and clinical treatment. The exclusion criteria consisted of the following: [1] patients who had received treatment or biopsy before the baseline CT examination, [2] patients diagnosed with large cavitary lung cancer, squamous carcinoma, SCLC, or pneumonia‐type ADC, and [3] lesions that had boundaries that could not be distinguished from consolidated lung tissue. A total of one hundred thirty‐three patients met the study criteria and were included in the analysis. Among these, 71 individuals were male, whereas 62 were female. The average age of the patients was 62.84 years, with a standard deviation of 11.62 years, ranging from 18 to 85 years. The cohort was divided into a training set comprising 93 cases and an independent test set containing 40 cases, maintaining a ratio of 7:3. Figure 1 represents the detailed patient recruitment process.

### 2.2 CT Image Acquisition

A spiral CT scanner was used to image each participant's chest (Somatom Force, Siemens, Germany). The scanning specifications were a field of view of 350 mm × 350 mm, a matrix size of 512 × 512, automatic tube current modulation, a slice thickness ranging from 1 to 1.5 mm, and a tube voltage of 120 kV. Every participant was administered an iodine contrast agent (60–85 mL) using a power syringe with a 2.5–3.0 mL/s rate. Venous and arterial phase CT images were obtained by rapid scanning, with a delay of 30 s for arterial and 60 s for venous images [22, 23].

### 2.3 Tumor Segmentation and Feature Analysis

Abnormalities were identified during examining arteries and veins at various stages of blood flow. The CT scans were segmented and analyzed for tumor characteristics using specific window levels: mediastinal window levels of 400 and 40 Hounsfield Units (HU) and lung window levels of 1600 HU and − 600 HU. Two blinded radiologists employed the RadCloud Platform (version 7.2; Huiying Medical Technology Co. Ltd.) for the semi‐automated segmentation of lesions on arterial phase CT images. This method ensured the omission of vessel margins from the contours. After segmentation of the arterial phase CT images, the resultant contours were applied to the venous phase images. Manual adjustments were implemented to correct misalignments caused by respiratory motion, ensuring precise alignment with venous phase contours.

To evaluate the reproducibility of the segmentation process, both intra‐observer and inter‐observer, two radiologists independently conducted the procedure on thirty selected cases one month apart. The consistency of findings among radiologists was evaluated using intra‐ and inter‐class correlation coefficients (ICCs). A senior radiologist subsequently assessed and confirmed each segmentation, addressing discrepancies through consensus.

The CT imaging findings were examined by the two radiologists, who agreed on each feature. The features assessed included intensity, lobulation, speculation, pleural retraction, vascular convergence, bronchial cutoff, and pleural effusion [17, 24, 25].

### 2.4 Radiomic Feature Extraction

This study followed the most recent recommendations for evaluating radiomics features set out by the Image Biomarker Standardization Initiative (IBSI) [26]. The RadCloud platform, which is constructed on top of the IBSI‐compliant PyRadiomics library [27] (v.3.1.0, https://pyradiomics.readthedocs.io/) in Python (v.3.7.0), was used to extract features. This library facilitates extracting a wide range of radiomics features from the original and filtered images. These features include Wavelet, Square, Gradient, Logarithm, Square Root, Exponential, and local binary patterns in 2D and 3D.

Before feature extraction, the CT images were normalized using PyRadiomics, and the voxel size was resampled to a standardized measurement of 1 × 1 × 1 mm^3^. The grayscale values were then transformed into discrete categories using a fixed bin width 25 [28]. The volume of interest (VOIs) for each patient in both the venous and arterial phases yielded 1688 radiomic features per phase. The features comprise 90 Neighboring Gray Tone Difference Matrix features, 432 Gray Level Co‐occurrence Matrix features, 252 Gray Level Dependence Matrix features, 288 Gray Level Run Length Matrix features, 288 Gray Level Size Zone Matrix features, and 14 3D Shape features. Considering the nearly identical morphological characteristics of venous and arterial phase VOIs, and after excluding 14 venous phase shape features, a total of 3362 features were collected from each patient.

### 2.5 Radiomic Feature Selection

Three processes were involved in selecting radiomic features from the training cohort, and the venous, arterial, and DPE‐CT features were each applied independently. Initially, ICCs were computed to evaluate the reproducibility of the features, while those with ICCs greater than 0.75 were kept [29]. The redundancy among the radiomic properties was then decreased using a Spearman correlation analysis. The least absolute shrinkage and selection operator (LASSO) Cox regression model was employed to determine the best features. The minimum 5‐fold cross‐validation error was the determining point for the regularization parameter λ in LASSO regression. A Cox proportional hazards regression model was employed to compute each patient's radiomics score (Rad‐score).

### 2.6 Model Development and Evaluation

Models were developed and evaluated employing radiomic characteristics, clinical risk variables, and a composite nomogram within the training cohort. A radiomics model (Rad model) was initially constructed using the Rad‐score obtained from univariate Cox analysis. The clinical model employed univariate Cox analysis to ascertain correlations between individual clinical features and PFS. Significant factors (*p* < 0.05) were used to develop a multivariate Cox model. Lastly, a nomogram was formulated by integrating the Rad‐score with clinical variables by multivariable Cox analysis. The nomogram's optimal cutoff value was obtained using the maximum selected rank statistics method, categorizing individuals into low‐ and high‐risk categories. The association between the nomogram and PFS was investigated using Kaplan–Meier survival analysis.

**FIGURE 2:**
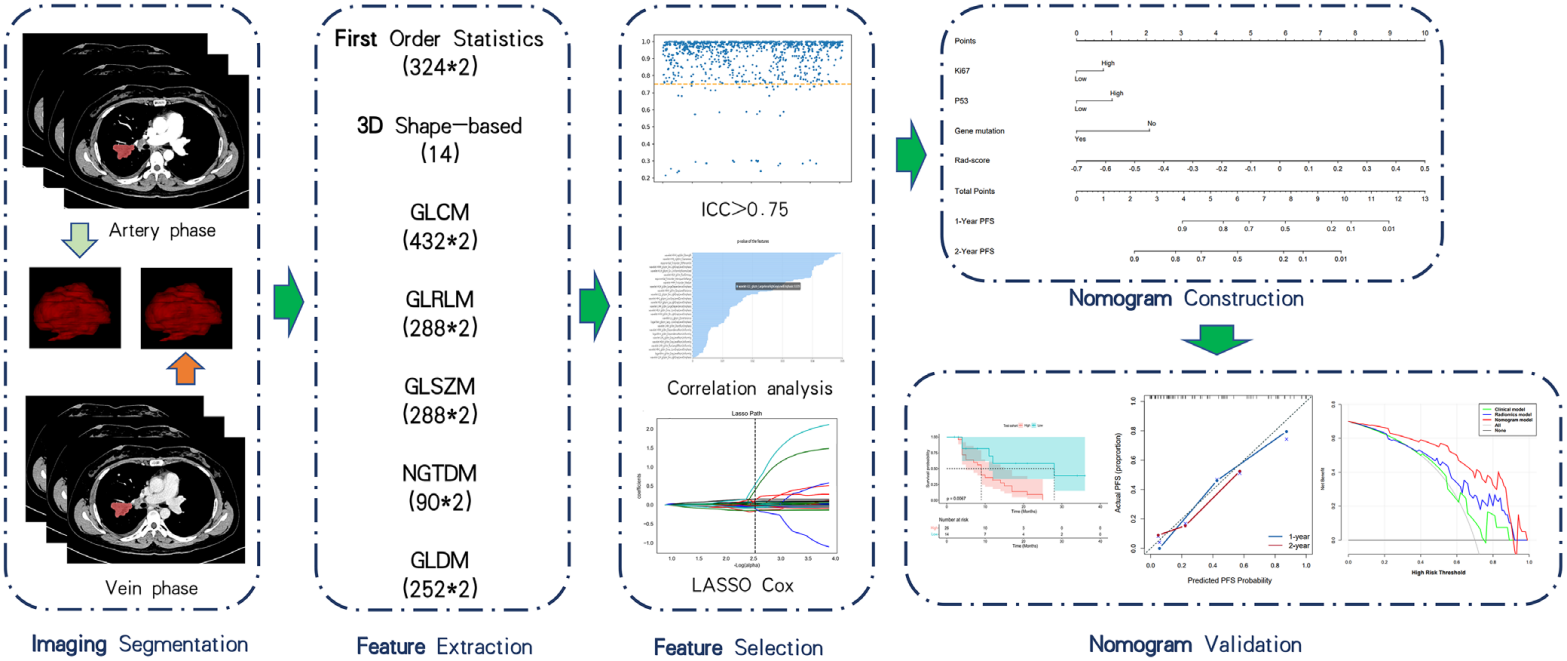
The study design. GLCM, gray‐level co‐occurrence matrix; GLDM, gray‐level dependence matrix; GLRLM, gray‐level run length matrix; GLSZM, gray‐level size zone matrix; ICC, intraclass correlation coefficients; LASSO, least absolute shrinkage, and selection operator; NGTDM, neighboring gray tone difference matrix.

Performance comparisons between the different models were conducted using Harrell's C‐index, decision curve analysis (DCA), integrated discrimination improvement (IDI), net reclassification improvement (NRI), and calibration curves. Figure 2 depicts the design of the flowchart for the study.

### 2.7 Statistical Analyses

Independent samples and categorical variables were subjected to chi‐square tests. The two data sets were compared using t‐tests for continuous variables. Independent predictive markers for PFS have been identified using univariate and multivariate Cox analysis. The efficiency of the nomogram was evaluated through the application of calibration curves. The log‐rank test and Kaplan–Meier survival analysis were utilized to evaluate the relationship between PFS and the radiomics nomogram (Rad‐nomogram). The evaluation of performance included DCA, NRI, and IDI. R (version 4.2.1; https://www.r‐project.org/) was employed for the statistical study. A two‐tailed p‐value was deemed statistically significant if it was lower than 0.05.

## 3 Results

### 3.1 Clinical Features

Table 1 presents the comprehensive imaging and clinical features of the test and training groups. Participants in the training group were monitored for a median of 8.0 months, with a range of 5.0 to 13.0 months. The median follow‐up duration for the test cohort was 9 months, with an interquartile range of 4.0 to 15.5 months. No discernible changes were observed between the two groups concerning PFS, imaging, or clinical features (*p* > 0.05).

**TABLE 1:** Clinical and imaging characteristics of patients in the training and test cohorts.

Characteristics	Total (*n* = 133)	Training (*n* = 93)	Test (*n* = 40)	*p*
Sex, *n* (%)				
Men	71 (53.4)	50 (53.8)	21 (52.5)	1.00
Female	62 (46.6)	43 (46.2)	19 (47.5)	
Age, Mean ± SD	62.84 ± 11.62	64.25 ± 11.23	59.58 ± 12	0.061
Smoking, *n* (%)				
No	80 (60.2)	57 (61.3)	23 (57.5)	0.829
Yes	53 (39.8)	36 (38.7)	17 (42.5)	
Family history of cancer, *n* (%)				
No	123 (92.5)	87 (93.5)	36 (90)	0.487
Yes	10 (7.5)	6 (6.5)	4 (10)	
Diabetes, *n* (%)				
No	112 (84.2)	74 (79.6)	38 (95)	0.084
Yes	21 (15.8)	19 (20.4)	2 (5)	
Hypertension, *n* (%)				
No	87 (65.4)	58 (62.4)	29 (72.5)	0.353
Yes	46 (34.6)	35 (37.6)	11 (27.5)	
Emphysema, *n* (%)				
No	108 (81.2)	74 (79.6)	34 (85)	0.622
Yes	25 (18.8)	19 (20.4)	6 (15)	
Homogeneous Intensity, *n* (%)				
No	87 (65.4)	62 (66.7)	25 (62.5)	0.791
Yes	46 (34.6)	31 (33.3)	15 (37.5)	
Lobulation, *n* (%)				
No	10 (7.5)	8 (8.6)	2 (5)	0.723
Yes	123 (92.5)	85 (91.4)	38 (95)	
Speculation sign, *n* (%)				
No	27 (20.3)	22 (23.7)	5 (12.5)	0.218
Yes	106 (79.7)	71 (76.3)	35 (87.5)	
Pleural retraction, *n* (%)				
No	28 (21.1)	20 (21.5)	8 (20)	1.00
Yes	105 (78.9)	73 (78.5)	32 (80)	
Bronchial cut‐off, *n* (%)				
No	76 (57.1)	53 (57)	23 (57.5)	1.00
Yes	57 (42.9)	40 (43)	17 (42.5)	
Vascular convergence, *n* (%)				
No	70 (52.6)	56 (60.2)	14 (35)	0.130
Yes	63 (47.4)	37 (39.8)	26 (65)	
Pleural effusion, *n* (%)				
No	98 (73.7)	66 (71)	32 (80)	0.384
Yes	35 (26.3)	27 (29)	8 (20)	
CEA, Median (Q1, Q3)	10.84 (4.87, 32.98)	10.84 (4.87, 32.98)	11 (4.68, 31.15)	0.646
NSE, Median (Q1, Q3)	17.28 (13.5, 22.3)	17.07 (12.94, 21.17)	18.68 (17.07, 28.17)	0.060
CA199, Median (Q1, Q3)	17.04 (10.24, 33.68)	17.04 (10.18, 37.73)	17.04 (10.69, 25.79)	0.834
Gene Mutation, *n* (%)				
No	71 (53.4)	51 (54.8)	20 (50)	0.746
Yes	62 (46.6)	42 (45.2)	20 (50)	
P53, *n* (%)				
Low	70 (52.6)	47 (50.5)	23 (57.5)	0.584
High	63 (47.4)	46 (49.5)	17 (42.5)	
Ki67, *n* (%)				
Low	74 (55.6)	51 (54.8)	23 (57.5)	0.926
High	59 (44.4)	42 (45.2)	17 (42.5)	
PFS, Median (Q1, Q3)	8 (4, 13)	8 (5, 13)	9 (4, 15.5)	0.680

Abbreviations: CA199, carbohydrate antigen 199; CEA, carcinoembryonic antigen; NSE, neuro‐specific enolase; PFS, progression‐free survival.

The results obtained from the univariate Cox regression analysis of the imaging and clinical features are compiled in Table 2. These findings revealed a strong correlation between PFS and gene mutation (hazard ratio [HR] = 0.515, *p* = 0.013), Ki67 (HR = 2.24, *p* = 0.001), and P53 (HR = 2.05, *p* = 0.005). Further, gene mutation (HR = 0.424, *p* = 0.002), Ki67 (HR = 2.226, *p* = 0.002), and P53 (HR = 1.920, *p* = 0.013) were independent prognostic factors for PFS, per the results of the multivariate Cox regression analysis (Table 3).

**TABLE 2:** Univariate Cox regression for predicting PFS in the training cohort.

Variable	HR	95% CI	*p*
Sex	0.921	0.563–1.51	0.745
Age	1	0.98–1.03	0.844
Smoking	0.685	0.402–1.17	0.165
Family	1.33	0.574–3.1	0.504
Diabetes	1.14	0.627–2.06	0.675
Hypertension	0.919	0.56–1.51	0.740
Emphysema	1.41	0.745–2.65	0.294
Homogeneous Intensity	1.5	0.856–2.64	0.156
Lobulation	2.67	0.801–8.9	0.110
Speculation sign	1.66	0.909–3.02	0.099
Pleural retraction	1.16	0.632–2.14	0.628
Bronchial cutoff	0.889	0.542–1.46	0.641
Vascular convergence	1.26	0.757–2.09	0.376
Pleural effusion	0.843	0.486–1.46	0.544
CEA	1.25	0.66–2.08	0.836
NSE	1.31	0.785–1.82	0.659
CA199	1.08	0.895–1.27	0.610
Gene mutation	0.515	0.304–0.873	0.013*
P53	2.05	1.23–3.41	0.005*
Ki67	2.24	1.36–3.67	0.001*

Abbreviations: CA199, carbohydrate antigen 199; CEA, carcinoembryonic antigen; CI, confidence interval; HR, hazard ratio; NSE, neuro‐specific enolase; PFS, progression‐free survival.

* *p* shows the value of univariate Cox regression analysis < 0.05.

**TABLE 3:** Multivariate Cox regression for predicting PFS in the training cohort.

Variable	B	SE	Z	*p* value	HR(95% CI)
Gene mutation	−0.858	0.277	−3.103	0.002*	0.424 (0.247–0.729)
P53	0.653	0.265	2.463	0.013*	1.920 (1.143–3.23)
Ki67	0.801	0.256	3.132	0.002*	2.226 (1.349–3.674)

Abbreviations: CI, confidence interval; HR, hazard ratio; PFS, progression‐free survival; SE, standard error.

* *p* shows the value of multivariate Cox regression analysis < 0.05.

### 3.2 Selected Radiomic Features and Development of Combined Radiomics Signature

The first step involved extracting 3362 radiomic features from the venous and arterial phase CT images. If the ICC was above 0.75, stable features were preserved and adjusted for further analysis. Following this, Spearman correlation analysis was employed to reduce redundancy among these radiomic characteristics. LASSO regression identified 9 nonzero coefficient radiomics features, with weights assigned as follows: 6 features from the arterial phase and 3 from the venous phase (Table 4). The Rad‐score was determined for each patient in the test and training groups based on the specified characteristics.

**TABLE 4:** The selected radiomics features of DPE‐CT and their corresponding coefficients.

Features	Coefficients
Wavelet‐LHH_glcm_Imc2_Artery	0.097449543
Wavelet‐HLH_glszm_SizeZoneNonUniformityNormalized_Vein	0.095857863
Lbp‐2D_firstorder_90Percentile_Artery	−0.019189956
Lbp‐2D_firstorder_RobustMeanAbsoluteDeviation_Artery	−0.03455879
Original_glszm_SizeZoneNonUniformity_Artery	0.031246948
Original_firstorder_Variance_Artery	0.04445144
Wavelet‐HLH_firstorder_TotalEnergy_Artery	0.037835382
Wavelet‐LHH_glszm_SmallAreaHighGrayLevelEmphasis_Vein	0.067209907
Wavelet‐HLH_glszm_HighGrayLevelZoneEmphasis_Vein	0.053230144

Abbreviation: DPE‐CT, dual‐phase enhanced computed tomography.

### 3.3 Rad‐Nomogram Development and Validation for PFS Prediction

Out of 1688 imaging characteristics from arterial and venous phases, 18 and 8 radiomic signatures were selected using a consistent methodology to establish the phase Rad‐score, respectively. Unlike the venous and arterial phase scores, the combined dual‐phase screening requires using distinct radiomic properties.

Table 5 presents the performance outcomes of the clinical, dual‐phase, venous phase, arterial phase, and nomogram models. The nomogram model better predicted PFS in both the training and test populations. The training group showed a C‐index of 0.794 (95% CI: 0.729–0.858), while the test cohort demonstrated a C‐index of 0.783 (95% CI: 0.699–0.868). The dual‐phase model demonstrated higher accuracy than the single venous‐phase, arterial‐phase, and clinical models.

**TABLE 5:** The Harrell's C‐index of study models for predicting PSF in the training and test cohorts.

Cohort	Model	C‐index	95% CI
Training			
	Clinical	0.688	0.621–0.756
	Artery phase	0.734	0.660–0.807
	Vein phase	0.696	0.619–0.773
	Dual‐phase	0.760	0.689–0.831
	Nomogram	0.794	0.729–0.858
Test			
	Clinical	0.675	0.551–0.798
	Artery phase	0.691	0.557–0.832
	Vein phase	0.678	0.553–0.786
	Dual‐phase	0.731	0.614–0.849
	Nomogram	0.783	0.699–0.868

Abbreviations: CI, confidence interval; HR, hazard ratio; PFS, progression‐free survival.

**FIGURE 3:**
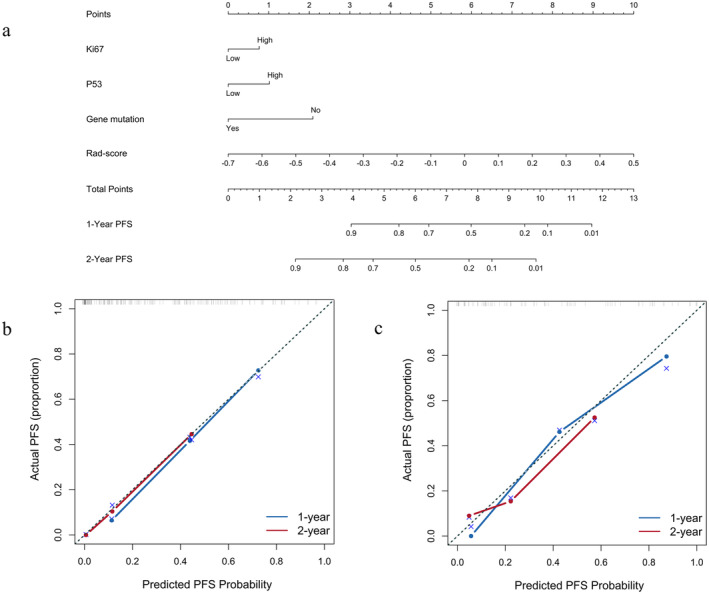
Development of the nomogram (a) for predicting PFS by combining Rad‐score, Ki67, P53, and gene mutation data. In both the test cohort (c) and the training group (b), the calibration profiles of the Rad‐nomogram for predicting PFS at the 1‐ and 2‐year time points demonstrated a satisfactory correlation. PFS, progression‐free survival.

This study introduced a Rad‐nomogram model for predicting PFS in individuals diagnosed with stage IV lung cancer. The nomogram combined clinical risk variables, particularly Ki67, P53, and gene mutation status, with radiomics features (Figure 3a). The calibration profiles for estimating the probability of PFS at 1 and 2 years showed a statistically significant relationship between the estimated probabilities and the actual observations in the training cohort (Figure 3b) as well as in the test cohort (Figure 3c).

**FIGURE 4:**
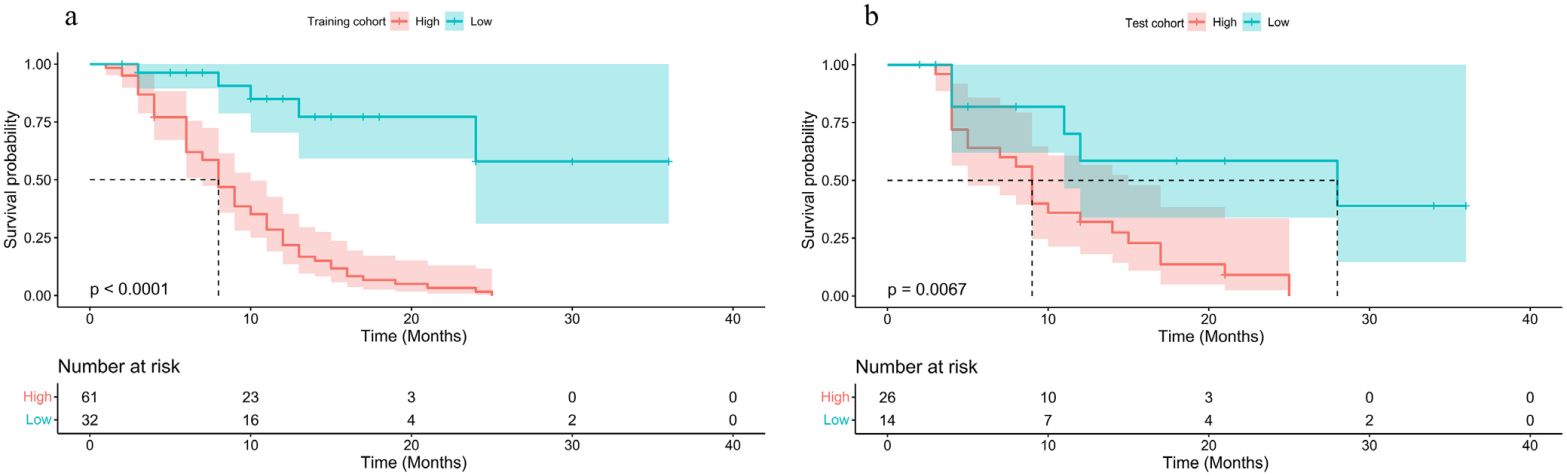
Kaplan–Meier curve analysis of the PFS between the high‐ and low‐risk groups in the training (a) and test groups (b), as determined by the Rad‐nomogram. PFS, progression‐free survival.

The Kaplan–Meier survival plot outcomes of the Rad‐nomogram are illustrated in Figure 4. Subsequent analysis revealed that the PFS of high‐risk patients was considerably lower than that of low‐risk patients in both the training (Figure 4a, *p* < 0.0001) and test groups (Figure 4b, *p* = 0.0067). The results validate the high prognostic accuracy of the Rad‐nomogram.

### 3.4 Models in DCA

**FIGURE 5:**
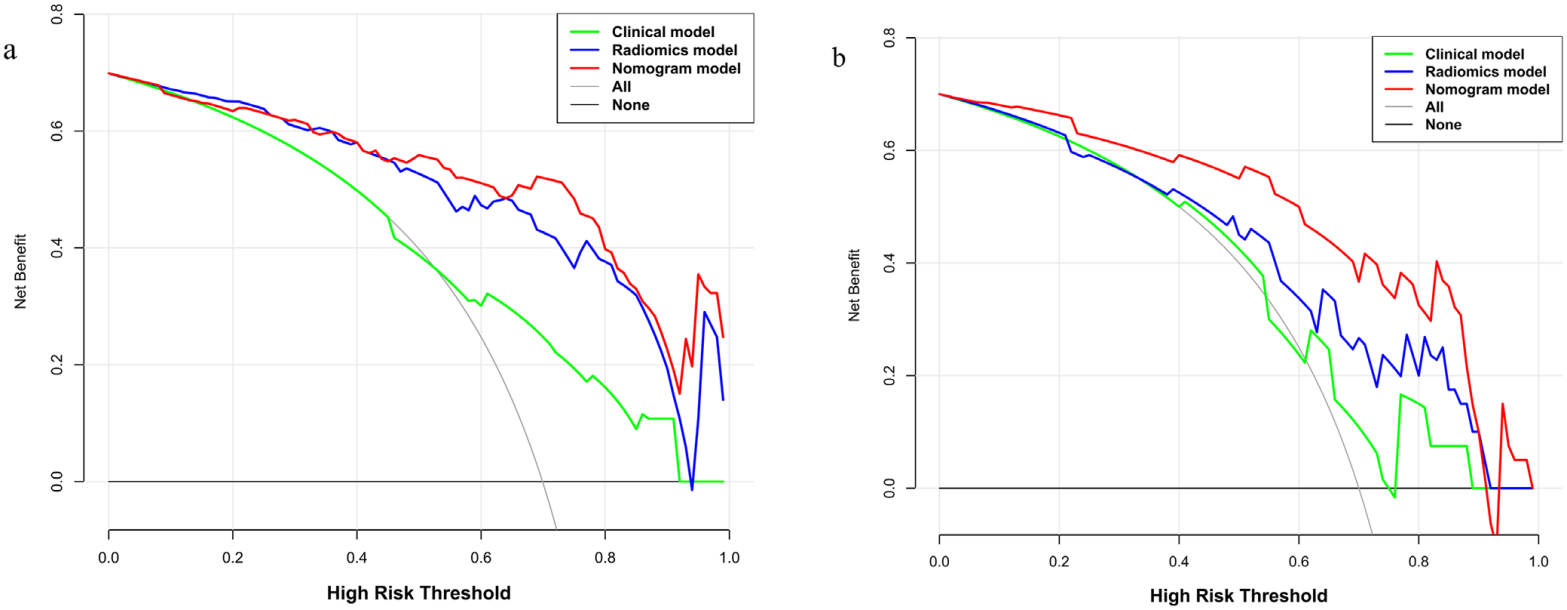
The DCA for every model in the test cohort (b) and training cohort (a). DCA, decision curve analysis.

A DCA comparing the Rad‐nomogram, clinical, and Rad models is shown in Figure 5. The data indicates that the Rad‐nomogram consistently yields a greater overall net benefit than both the clinical and Rad models over most potential threshold probabilities in both the training (Figure 5a) and test groups (Figure 5b). Table 6 further details the NRI and IDI for the Rad‐nomogram in both of the cohorts. In the training cohort, the nomogram model achieved an NRI of 46.6% and 31.6% and an IDI of 22.1% and 5.8%, respectively, compared to the clinical and Rad models (*p* < 0.05). In the test group, the nomogram indicated a NRI of 52.6% and 46.7% and an IDI of 20.7% and 11.6% (*p* < 0.05). The radiomics and clinical models demonstrated NRI and IDI values of 6.4% and 9.1% in the test cohort, respectively; however, these differences were not statistically significant (*p* > 0.05).

**TABLE 6:** The clinical benefits and utility of nomogram compared with clinical and Rad models.

Model	NRI (95%CI)	*p*	IDI (95%CI)	*p*
Training cohort				
Nomogram vs. Clinical	0.466 (0.024–0.654)	< 0.001	0.221 (0.019–0.381)	< 0.001
Nomogram vs. Radiomics	0.316 (0.030–0.632)	< 0.001	0.058 (0.000–0.107)	< 0.001
Radiomics vs. Clinical	0.176 (−0.107–0.465)	0.182	0.163 (0.008–0.235)	< 0.001
Test cohort				
Nomogram vs. Clinical	0.526 (0.039–0.321)	< 0.001	0.207 (0.030–0.382)	0.020
Nomogram vs. Radiomics	0.467 (0.054–0.706)	< 0.001	0.116 (0.027–0.774)	0.040
Radiomics vs. Clinical	0.064 (−0.487–0.598)	0.773	0.091 (−0.223–0.299)	0.574

Abbreivations: CI, confidence interval; IDI, integrated discrimination improvement; NRI, net reclassification.

## 4 Discussion

The present study demonstrated that a Rad‐nomogram, integrating imaging radiomics features and clinical characteristics, can effectively identify stage IV lung ADC patients with poor PFS. A thorough evaluation of several models for PFS prediction was also carried out, including the nomogram, artery phase Rad model, vein phase Rad model, dual‐phase Rad model, and clinical model. The dual‐phase Rad model produced outstanding results compared to the single‐phase and clinical models. Furthermore, the Rad‐nomogram, which uses DPE‐CT imaging, provided patients receiving systemic treatment with personalized estimates of the probability of progression.

In recent years, targeted, immune‐based, and advanced chemotherapeutic drugs in individualized therapy for ALC have produced encouraging patient outcomes. However, the primary challenge continues to be the development of drug resistance and treatment failure, which are ascribed to various unclear and unknown factors [30, 31]. Therefore, there are significant clinical implications for personalized therapy scheduling when predicting the PFS of ALC patients using preoperative CT imaging. Obtaining many intricate and retrievable features from medical imaging data is known as “radiomics.” These characteristics can be obtained and examined separately or with clinical prognostic variables. Radiomics has shown greater scientific potential for medical research [10, 32, 33]. Several publications verify that radiomics aids in predicting the therapeutic outcome of ALC patients undergoing targeted therapy, immunosuppressant therapy, or chemotherapy. Previous studies predominantly employed a single‐phase enhanced CT or clinical model to predict therapeutic outcomes [11, 12, 34, 35].

The current study used DPE‐CT imaging and clinical models, differing from previous research. The results demonstrated that the artery phase was similar to the vein phase in predicting PFS for patients with stage IV lung ADC in the testing cohort (C‐index, 0.691 and 0.678). The results are consistent with data from previous research indicating comparable predictive efficacy for therapeutic outcomes when employing both vein and artery phase CT images [16]. Chen et al. previously extracted features from single enhanced mediastinal window and lung window images to predict PFS in patients with SCLC [12]. However, there has been a lack of assessment of the effectiveness of DPE‐CT.

Furthermore, the present study discovered that combined DPE‐CT yielded better predictive results for PFS than single‐phase models (C‐index, 0.731). Prior studies have revealed that radiomics based on multi‐phase enhanced CT images are more efficient than single‐phase ones in predicting gene mutations and histopathology subtypes of lung cancer [14, 15], validating the current results. This highlights the potential of using extensive radiomic characteristics obtained from DPE‐CT scans to improve the accuracy of predictions.

According to this study, P53, Ki67, and gene mutations are crucial markers for predicting PFS after systemic treatment. This was determined using both univariate and multivariate Cox regression analysis. Previous research has given limited attention to the role of immunohistochemical indicators in clinical feature‐based nomogram predictions. Higher expression levels of Ki67 and P53 are generally associated with poorer therapeutic efficacy in ALC [36, 37]. Moreover, molecularly targeted therapeutics have improved PFS in patients with gene mutations [4]. The present study developed a clinical prediction model for PFS, which demonstrated a C‐index of 0.675 in the test group, aligning with previous findings.

A nomogram prediction model was developed that combines dual‐phase radiomics signatures and clinical factors, demonstrating improved accuracy in predicting PFS with a C‐index equal to 0.783 within the test group. It was suggested that this Rad‐nomogram successfully classified stage IV lung ADC patients into high‐ and low‐risk groups based on the Kaplan–Meier curves that showed significant variations in PFS. Based on the findings of previous research studies, nomograms that combine several prognostic signs can predict the prognosis of lung cancer [34, 35]. The present findings suggest that DPE‐CT image‐based radiomics and clinical models are complementary, and their combination offers a more accurate prediction of PFS.

In survival analysis, the clinical application of the Rad‐nomogram was rigorously evaluated through the application of DCA, NRI, and IDI, as commonly employed in prior investigations [17, 38, 39]. The current results demonstrated that the DCA for the Rad‐nomogram, which integrates radiomics and clinical aspects, demonstrated improved clinical application in comparison to both clinical and Rad models alone, which is in line with other studies. Furthermore, adding radiomics features to the clinical model led to a substantial rise in both NRI and IDI, which ranged from 11.6% to 52.6% in the test cohort, supporting these findings even more. This significant improvement highlights the nomogram's increased PFS classification accuracy and predictive efficiency. However, it is noteworthy that while positive NRIs and IDIs (6.4% and 9.1%, respectively) were observed between the radiomics and clinical model, significant differences were absent (*p* > 0.05) in the test cohort. These findings further demonstrated that the complementarity of radiomics and clinical characteristics can considerably enhance the prediction of PFS in advanced lung ADC.

The current study is subject to several limitations. The applicability of the resulting models may be limited by the study's retrospective nature and its dependence on a singular central dataset, which lacks external validation. Furthermore, the restricted sample size may impact the statistical robustness of the analysis. Thirdly, the predictive accuracy of the models may have been affected by the heterogeneity in therapeutic schedules among the patients. Moreover, the exclusion of non‐enhanced CT scanning protocols due to data incompleteness raises concerns about the impact of contrast enhancement on the performance of Rad models. This area continues to be a subject of debate. Therefore, it is necessary to conduct future research that uses multiphasic contrast‐enhanced CT imaging to accurately predict clinical outcomes in advanced lung ADC and get more clarification on the subject.

In summary, the outcomes of the study emphasize the significance of a nomogram that combines a clinical model with a Rad model based on DPE‐CT to predict PFS in patients with stage IV lung ADC. This risk stratification, determined from a nomogram, reveals a significant potential to improve customized treatment methods for individuals with ALC.

## Author Contributions

**Haitao Sun:** conceptualization (equal), funding acquisition (equal), investigation (equal), methodology (equal), visualization (equal), writing – original draft (equal). **Zhaohui Peng:** data curation (equal), methodology (equal), resources (equal). **Guoyue Chen:** data curation (equal), methodology (equal), resources (equal). **Zhengjun Dai:** formal analysis (equal), software (equal), visualization (equal). **Jian Yao:** conceptualization (equal), funding acquisition (equal), resources (equal). **Peng Zhou:** conceptualization (equal), investigation (equal), methodology (equal), project administration (equal), supervision (equal), writing – review and editing (equal).

## Ethics Statement

Approval was obtained from the Ethic committee of Central hospital affiliated to Shandong First Medical University. The procedures used in this study adhere to the tenets of the Declaration of Helsinki.

## Consent

Since this study was a retrospective study, the Ethic committee of Central hospital affiliated with Shandong First Medical University waived the need to obtain informed consent from the patients.

## Conflicts of Interest

The authors declare no conflicts of interest.

## Data Availability

The datasets generated during and/or analyzed during the current study are available from the corresponding author on reasonable request.
